# Frontotemporal Dementia and Glucose Metabolism

**DOI:** 10.3389/fnins.2022.812222

**Published:** 2022-02-23

**Authors:** Liam Rodney Garrett, Teresa Niccoli

**Affiliations:** Genetics, Evolution and Environment Department, Institute of Healthy Ageing, University College London, London, United Kingdom

**Keywords:** FTD, glucose, TDP-42, FUS, MAPT, C9orf72, C9orf72 ALS/FTD

## Abstract

Frontotemporal dementia (FTD), hallmarked by antero-temporal degeneration in the human brain, is the second most common early onset dementia. FTD is a diverse disease with three main clinical presentations, four different identified proteinopathies and many disease-associated genes. The exact pathophysiology of FTD remains to be elucidated. One common characteristic all forms of FTD share is the dysregulation of glucose metabolism in patients’ brains. The brain consumes around 20% of the body’s energy supply and predominantly utilizes glucose as a fuel. Glucose metabolism dysregulation could therefore be extremely detrimental for neuronal health. Research into the association between glucose metabolism and dementias has recently gained interest in Alzheimer’s disease. FTD also presents with glucose metabolism dysregulation, however, this remains largely an unexplored area. A better understanding of the link between FTD and glucose metabolism may yield further insight into FTD pathophysiology and aid the development of novel therapeutics. Here we review our current understanding of FTD and glucose metabolism in the brain and discuss the evidence of impaired glucose metabolism in FTD. Lastly, we review research potentially suggesting a causal relationship between FTD proteinopathies and impaired glucose metabolism in FTD.

## Introduction

Frontotemporal dementia (FTD), indicated by neurodegeneration in the antero-temporal lobes of the brain, is the second most common early onset dementia affecting ∼3/100,000 individuals under the age of 65 (De Conti et al., 2017). Approximately 40% of FTD patients have a family history of dementia, suggesting strong heritability (De Conti et al., 2017). Nonetheless, the pathophysiology of FTD remains largely unknown (Liu et al., 2019).

### Introduction to Frontotemporal Dementia Pathology

Clinically there are two major forms of FTD, termed behavioral variant (bhv)-FTD and primary progressive aphasia (PPA)-FTD, which is further divided into semantic (sv)-FTD and non-fluent (nfv)-FTD (Liu et al., 2019). These three variants of FTD show different symptoms that are associated with neurodegeneration in distinct regions of the brain (Table 1; Borroni et al., 2012; Liu et al., 2019). Notably, FTD and amyotrophic lateral sclerosis (ALS) are considered to be neurodegenerative diseases lying on one spectrum, sharing pathological and genetic characteristics, with nearly 50% of ALS patients co-presenting with FTD (Lomen-Hoerth et al., 2003; Ling et al., 2013). Additionally, FTD cases may co-present with corticobasal syndrome or progressive supranuclear palsy, both being forms of parkinsonism (Kertesz and Munoz, 2004; Josephs et al., 2006; Hassan et al., 2012).

**TABLE 1:** Summary of the different clinical forms of FTD and their relationships to presentation, pathology and genetics.

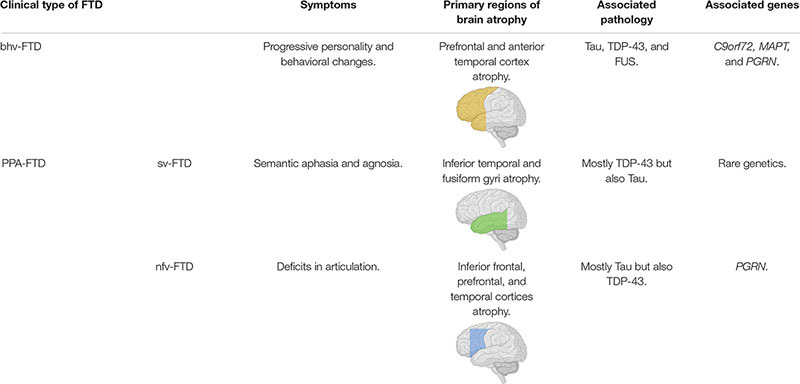

*Associated brain images depict primary regions of brain atrophy for associated clinical FTD forms. Images created with: BioRender.com.*

FTD is pathogenically diverse; different patients diagnosed with the same clinical form of FTD may present with distinct pathologies (Kertesz et al., 2005; Snowden et al., 2015), characterized by the presence of misfolded protein aggregates (proteinopathies) in affected regions of the brain—where deposition patterns may vary across clinical forms (De Conti et al., 2017; Tsai et al., 2019). To date, four major proteinopathies have been identified in FTD patients: Tau, TAR DNA-binding protein 43 (TDP-43), fused in sarcoma (FUS), and ubiquitin positive inclusions, the latter comprising unknown aggregated proteins; with the first two accounting for the vast majority of cases (Neumann et al., 2009; MacKenzie et al., 2010).

Tau, TDP-43 and FUS are all highly abundant in brains, comprising intrinsically disordered domains, and the latter two perform similar functions, however, it’s not clear whether they all converge on a single pathogenic cascade.

TARDP encodes TDP-43, a heterogeneous nuclear ribonucleoprotein (hnRNP), that possesses the ability to shuttle in-between the nucleus and the cytoplasm but primarily localizes to the nucleus (Ayala et al., 2008; Barmada et al., 2010; Jo et al., 2020). It binds RNA in a sequence-specific manner and regulates mRNA metabolism, including nuclear mRNA processing, mRNA stability and mRNA transport. Rarely, it carries pathogenic FTD mutations (Strong et al., 2007; Mackenzie et al., 2010; Ayala et al., 2011; Buratti and Baralle, 2012). TDP43 aggregates, as well as being a common pathological hallmark in FTD, are often found in motoneurons of ALS patients. However, its pathophysiology remains mostly unknown (Neumann et al., 2006). TDP-43 knockout is embryonically lethal and conditional knockout of TDP-43 yields motor neuron defects in mice, suggesting a possible loss of function model in FTD-TDP-43 (Wu et al., 2009; Iguchi et al., 2013). Nonetheless, a gain of toxicity model can’t be excluded. Transfecting TDP-43 inclusions reduces neuroblastoma cell viability and over-expression of TDP-43 in cell and animal models leads to phenotypes analogous to those found in patients (Estes et al., 2011, 2013; Capitini et al., 2014).

Several mutations in other genes have also been linked to TDP-43 pathology (Table 2). A hexanucleotide repeat expansion in chromosome 9 open reading frame 72 (C9) is the most common genetic cause of FTD and is associated with ∼10% of all FTD patients in Western Europe (van der Zee et al., 2013). C9 mutations lead to TDP-43 proteinopathy possibly by stopping it from shuttling into the nucleus, causing its cytoplasmic accumulation and aggregation (Cook et al., 2020). Mutations to Progranulin (*PGRN*) are found in 5–10% of all FTD cases, and *PGRN* mRNA depletion causes TDP-43 cleavage and subsequent inclusion formation (Gass et al., 2006; Zhang et al., 2007). Finally, mutations to valosin-containing protein (*VCP*) have been associated with FTD-TDP-43 pathology with *VCP* mutants preventing autophagosome maturation, which may lead to the increased inclusion of TDP-43 by preventing its degradation (Gitcho et al., 2009; Ju et al., 2009).

**TABLE 2:** List of some of the most commonly reported mutations associated with FTD.

Gene	Identified mutations linked to FTD
*MAPT*	R5H, R5L, K257T, I260V, L266V, G272V, N279K, ΔK280, L284L, ΔN296, N296N, N296H, P301L, P301S, G304S, S305N, S305S, L315R, S320F, Q336R, V337M, E342V, S352L, V363I, K369I, G389R, R406W (Goedert and Jakes, 2005)
*PGRN*	M1?, W7R, A9D,R19W, D22R, C31L, N33N, G35E, T52H, L53P, G79D, S82V, F86S, E88E, A89V, C105R, ΔR110, R110Q, N118F, S120Y, V121W, I124T, ΔQ125, P127R, N128N, Q130S, A155W, C157K, G168S, R177H, T182M, A199V, V200G, S203V, S226W, P233Q, N236N, A237W, P248L, ΔC253, S258N, L271L, T272S, V279G, E287D, ΔQ300, A303G, W304G, W304L, ΔW304, V279G, S301S, ΔC314, A323T, G333V, ΔQ337, P357H, ΔC366, T382S, T382N, ΔW386, ΔQ401, V411S, ΔA412, ΔQ415, ΔR418, R418Q, R432C, R433W, R433Q, C466L, ΔC468, L469F, C474C, ΔR493, G515A, ΔR535, W541C (Gass et al., 2006; Gijselinck et al., 2008)
*C9ORF72*	GGGGCC hexanucleotide repeat expansion in intron 1 (DeJesus-Hernandez et al., 2011)
*TARDP*	N267S, A382T, K263E (Huey et al., 2012)
*FUS*	P106L, ΔG174-G175, Q179H, R521H (Broustal et al., 2010; Huey et al., 2012)
*CHMP2B*	C-terminal deletion mutations including ΔQ165 (Isaacs et al., 2011)
*VCP*	R93G, R95G, R95H, R155H, R155C, R155P, R159C, R191Q, L198W, A232E, N387H (Watts et al., 2007; Gitcho et al., 2009)

FUS is also an hnRNP and fulfills similar roles to TDP-43 (De Conti et al., 2017). FTD-FUS pathophysiology is still unclear, with some experimental support for both gain and loss of function models (De Conti et al., 2017). Notably, FUS knockout mice have no motor neuron defects and mutations in FUS have only rarely been found in FTD-FUS (Seelaar et al., 2010; Huey et al., 2012; Sharma et al., 2016), although they are relatively common in familial ALS (Blair et al., 2010; Mejzini et al., 2019).

FTD-Tau, accounts for 40% of all FTD cases and is characterized by the presence of cytosolic, hyperphosphorylated, insoluble filaments of the microtubule-associated protein Tau (*MAPT*) in neurons and glia (Tauopathy) (Liu et al., 2019). Tau modulates microtubules stability by promoting tubulin polymerization and exists in six isoforms that are equally expressed in healthy subjects and differ by their affinity to microtubules (Goedert et al., 1989; Barbier et al., 2019). Tau is regulated by post-translational modifications; hyperphosphorylation decreases Tau’s microtubule binding affinity and promotes aggregate formation (Kuret et al., 2005). Most FTD-Tau cases have no direct genetic cause, with mutations in *MAPT* accounting for less than 25% of cases, (Rohrer and Warren, 2011). Over 50 *MAPT* mutations (as of 2018) have been identified (Table 2), with diverse pathogenic roles. A significant portion of pathogenic *MAPT* mutations reside within intron 10 and cause an increase in 4R Tau isoform, by interfering with mRNA processing (Strang et al., 2019). Others, for example, impair Tau’s interaction with protein phosphatase 2A (PP2A), preventing its dephosphorylation (Goedert et al., 2002; Strang et al., 2019). However, the molecular pathophysiology behind FTD-Tau remains largely unknown (De Conti et al., 2017).

FTD-UPS contains an aggregating protein of unknown identity (MacKenzie et al., 2010). This has made research into FTD-UPS pathophysiology difficult. Mutations in the charged multivesicular body protein 2B (*CHMP2B*; encoding a protein involved in protein sorting and trafficking), observed in familial FTD cases in Denmark and Belgium, leads to FTD-UPS (Isaacs et al., 2011).

### Introduction to Brain Glucose Metabolism

Glucose metabolism is paramount in the brain. The brain has a particularly high energy demand, accounting for 20% of the body’s energy consumption, but only 2% of its mass, this energy demand is mostly met by the metabolism of glucose (Herculano-Houzel, 2011).

**FIGURE 1:**
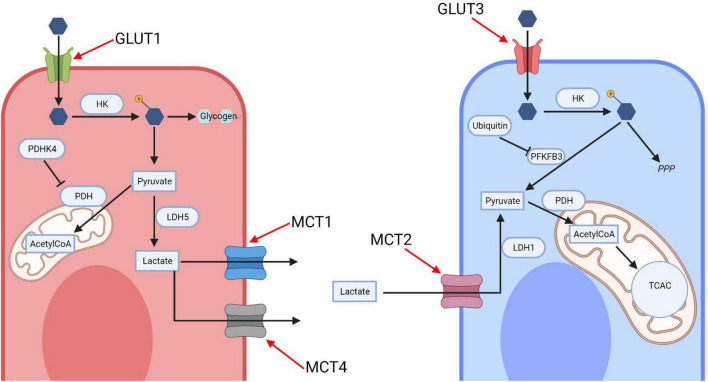
The astrocyte-neuron lactate shuttle (ANLS). Glucose is imported into neurons and astrocytes by their respective transporters, GLUT1 and GLUT3, before being phosphorylated by hexokinase. In neurons most glucose-phosphate is subsequently metabolized along the pentose phosphate pathway, with the early glycolytic enzyme PFKFB3 being inhibited by ubiquitin action. In astrocytes glucose-phosphate is converted into glycogen for storage or metabolized *via* glycolysis to produce pyruvate, which either enters the mitochondria or is converted into lactate by lactate dehydrogenase (LDH) 5. Lactate is exported by the monocarboxylate transporters (MCT) 1 and 4 and imported into neurons *via* the MCT 2. It is then converted back to pyruvate *via* LDH-1 and enters the mitochondrial oxidative phosphorylation cycle. HK, hexokinase; PDHK4, pyruvate dehydrogenase kinase 4; LDH, lactate dehydrogenase; MCT, monocarboxylate transporter; PDH, pyruvate dehydrogenase; TCAC, tricarboxylic acid cycle; PFKFB3, 6-phosphofructo-2-kinase/fructose-2,6-biphosphatase 3; PPP, pentose phosphate pathway; AcetylCoA, Acetyl coenzyme A. Created with: BioRender.com.

**FIGURE 2:**
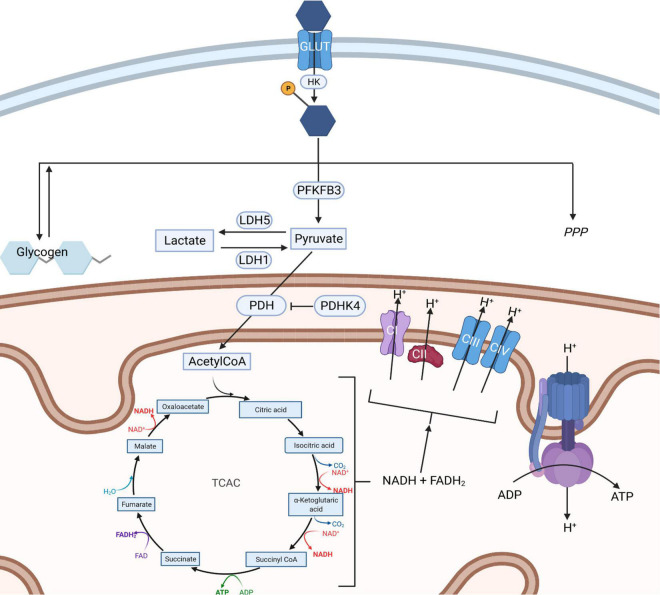
Routes of glucose metabolism in cells. Upon entering a cell *via* a glucose transporter (GLUT) glucose is phosphorylated by hexokinase entrapping glucose-phosphate. This is then metabolized into glycogen (left), along the pentose phosphate pathway (right) or *via* glycolysis (center). Glycolysis results in the formation of pyruvate which can be interconverted into lactate through the action of lactate dehydrogenase enzymes. Pyruvate can also enter mitochondria, where it is converted into AcetylCoA through the action of pyruvate dehydrogenase which in turn fuels the tricarboxylic acid cycle (TCAC). The TCAC cycle generates NADH and FADH_2_ which are required to supply electrons to the electron transport chain (ETC). The ETC generates a proton gradient across the inner mitochondrial membrane, which is used to power the production of ATP by complex V, an ATP synthase. HK, hexokinase; PDHK4, pyruvate dehydrogenase kinase 4; LDH, lactate dehydrogenase; MCT, monocarboxylate transporter; PDH, pyruvate dehydrogenase; TCAC, tricarboxylic acid cycle; PFKFB3, 6-phosphofructo-2-kinase/fructose-2, 6-biphosphatase 3; PPP, pentose phosphate pathway; ETC, electron transport chain; AcetylCoA, Acetyl coenzyme A; NADH, nicotinamide adenine dinucleotide; FADH_2_, flavin adenine dinucleotide; ATP, Adenosine triphosphate; ADP, Adenosine diphosphate. Created with: BioRender.com.

Glucose uptake into the brain and its cells is mediated by the glucose transporter (GLUT) family. GLUT1 is responsible for glucose transport across the blood brain barrier and into glia, whilst GLUT3 mediates neuronal glucose uptake (Figure 1; Koepsell, 2020). Glucose metabolism yields energy in the form of Adenosine-5′-triphosphate (ATP; the body’s energy substrate) and precursors for neurotransmitter synthesis, nucleic acid and protein production (Magistretti and Allaman, 2018). Glucose is metabolized through glycolysis and the tricarboxylic acid cycle (TCAC; Figure 2), or the pentose phosphate pathway (PPP) (Camandola and Mattson, 2017). Upon import, glucose is converted into glucose-6-phosphate by hexokinase, which traps it inside the cell (Magistretti and Allaman, 2018). Glucose-6-phosphate subsequently enters either the PPP, the glycolysis pathway, or it is converted into glycogen (an energy store in the brain) (Brown, 2004; Magistretti and Allaman, 2018). The PPP converts glucose-6-phosphate into products crucial for maintaining the cell’s redox homeostasis and produces precursors for nucleic acid and sugar phosphate synthesis (Camandola and Mattson, 2017). Glycolysis metabolizes glucose into pyruvate which is transported into mitochondria where it is converted into Acetyl coenzyme A (acetyl-CoA), which enters the TCAC (Camandola and Mattson, 2017; Magistretti and Allaman, 2018). This, through a series of reactions, produces reduced nicotinamide adenine dinucleotide (NADH) and flavin adenine dinucleotide (FADH_2_) (Camandola and Mattson, 2017). These products are subsequently re-oxidized through the electron transport chain, generating a proton gradient which drives hydrogen import into the intermembrane space *via* ATP synthase, generating ATP, a process termed oxidative phosphorylation (Camandola and Mattson, 2017).

The brain’s glucose metabolism is coupled between neurons and glia *via* an astrocyte-neuron lactate shuttle (ANLS) (Magistretti and Allaman, 2018). Astrocytes promote glycolytic lactate production and downregulate mitochondrial oxidation (Figure 1; Magistretti and Allaman, 2018). High expression levels of pyruvate dehydrogenase kinase 4 (PDK4) in astrocytes cause the inhibition of pyruvate dehydrogenase (PDH, the enzyme converting pyruvate into acetyl-CoA pyruvate into acetyl-CoA), leading to a drop in acetyl-CoA production and therefore of the TCAC cycle (Zhang et al., 2014). Furthermore, astrocytes mostly favor lactate production by predominantly expressing lactate dehydrogenase (LDH)-5, which promotes lactate production (Bittar et al., 1996). Additionally, astrocytic mitochondrial electron transport chain complex I is uncoupled, reducing oxidative phosphorylation energy yield; mice with inhibited astrocytic mitochondrial respiration remain phenotypically normal as do flies (Volkenhoff et al., 2015; Lopez-Fabuel et al., 2016).

Neurons are hypothesized to preferentially metabolize glucose through the PPP, to maintain their redox homeostasis and prevent oxidative stress (Magistretti and Allaman, 2018). Neurons establish this PPP preference by lowering the glycolytic enzyme 6-phosphofructo-2-kinase/fructose-2,6-biphosphatase 3 through its continual degradation (Herrero-Mendez et al., 2009). Thus, neurons require an alternative source for energy production—lactate; and neurons preferentially consume lactate over glucose as an energy source in culture (Bouzier-Sore et al., 2003). Neurons also express less PDK4, thus not inhibiting PDH, and express LDH-1 which favors pyruvate synthesis (Bittar et al., 1996; Zhang et al., 2014).

The ANLS model proposes that astrocytes fuel neuronal energy demands by transferring lactate to neurons (Figure 1), which can be metabolized very quickly (Magistretti and Allaman, 2018). This system is therefore able to sustain neurons’ dynamic energy demands, where neurons can rely on a continuous lactate supply when their energy demand rises rapidly during times of increased neuronal excitation (Mason, 2017). The shuttles’ existence is supported by the expression patterns of monocarboxylate transporters (MCT) in astrocytes and neurons. Astrocytes predominantly express MCT1 and MCT4, which have relatively low lactate affinity compared to the neuronally expressed MCT2, hence promoting unidirectional lactate transport (Figure 1; Pierre and Pellerin, 2005). Astrocytes increase their glucose consumption more than neurons during high energy demand, even though neurons have greater energy requirements (Chuquet et al., 2010). Supporting this, selective knockdown of *Drosophila melanogaster* astrocytes’ glycolytic enzymes (but not neuronal ones) leads to neurodegeneration (Volkenhoff et al., 2015).

Most cellular processes are directly linked to glucose metabolism. Rapid drops in ATP concentrations can cause necrosis and apoptosis, and processes required for protein and organelle turn-over are energy dependent, as is intracellular transport of products including mRNAs (Zhao et al., 2008). Hence it is unsurprising that many neurodegenerative diseases, including FTD, not only share the formation of protein aggregates, failed protein degradation and cell death, but also impaired glucose metabolism.

## Impaired Glucose Metabolism in Frontotemporal Dementia Patients

Glucose metabolism can be observed in brains using the radioactive glucose analog tracer 18F-fluorodeoxyglucose (FDG) in positron emission tomography (PET); a process termed FDG-PET (Meeter et al., 2017). FDG-PET allows to visualize areas with divergent relative uptake of FDG and therefore glucose in brains, allowing to evaluate glucose metabolism (Meeter et al., 2017). FDG-PET has highlighted the divergent patterns of glucose utilization across FTD subtypes (Meeter et al., 2017).

Brain glucose hypometabolism is observed across the FTD spectrum (Desgranges et al., 2007; Rabinovici et al., 2008; Deters et al., 2014; Morbelli et al., 2016). In bhv-FTD, a study identified glucose hypometabolism in the cortical regions (including the frontal and anterior temporal areas, cingulate gyri, uncus, and insula) and subcortical areas (including the basal ganglia and medial thalamic region) (Jeong et al., 2005). Glucose hypometabolism in sv-FTD was primarily observed in the anterior temporal lobes. Studies with 8–10 patients identified glucose hypometabolism extended into the right caudate nucleus, insula and bilateral orbitofrontal areas, however, in one study, participants all had advanced sv-FTD, which may explain the more extensive glucose hypometabolism (Diehl-Schmid et al., 2006; Desgranges et al., 2007). Nfv-FTD patients display larger heterogeneity in patterns of glucose hypometabolism; with the inferior frontal gyrus, the dorsolateral frontal cortex, the anterior cingulate cortex and the insula being primarily affected by glucose hypometabolism (Cerami et al., 2017). Interestingly, across all clinical subtypes, a pattern of glucose hypometabolism favoring the left hemisphere of the brain was identified (Jeong et al., 2005; Diehl-Schmid et al., 2006; Desgranges et al., 2007; Rabinovici et al., 2008; Acosta-Cabronero et al., 2011; Bejanin et al., 2020). Although, in the case of bhv-FTD this is contested with one study identifying bilateral glucose hypometabolism (Diehl-Schmid et al., 2007).

Familial FTD FDG-PET studies similarly identified glucose hypometabolism across subtypes with patterns mainly aligning to the clinical subtype of individual patients (Castelnovo et al., 2019; Clarke et al., 2021). *PGRN* mutation carriers, displayed large heterogeneity in their glucose hypometabolism patterns in studies with 9–10 patients, aligning with the fact that *PGRN* mutations cause FTD across the clinical spectrum (Jacova et al., 2013; Licata et al., 2020). Nevertheless, one study found that ∼80% of *PGRN* mutation carrying patients had glucose hypometabolism in the temporal regions of the brain, which extended beyond the boundaries of the frontotemporal region (Licata et al., 2020). C9 carriers were found to have glucose hypometabolism extending into the cerebellar cortex, occipital cortex, cingulate cortex, rolandic operculum, and caudate nuclei (Castelnovo et al., 2019; Diehl-Schmid et al., 2019). Furthermore, C9 carriers also showed a characteristic thalami glucose hypometabolism (Diehl-Schmid et al., 2019). With over 70 FTD causal *MAPT* pathogenic mutations known to date, glucose hypometabolic patterns have been harder to establish across *MAPT* mutation carriers (Clarke et al., 2021). P301L *MAPT* mutation carriers were found to develop glucose hypometabolism in the anterior cingulate, aligning with their clinical presentation of bhv-FTD (Clarke et al., 2021). By contrast, N279K *MAPT* mutation carriers in a study with 5 patients had asymmetrical glucose hypometabolism in the temporal, frontal and parietal lobe (which in some cases extended into the thalami and basal ganglia) (Arvanitakis et al., 2007).

Collectively, these studies identify significant glucose hypometabolism in FTD patients, with identifiable patterns across subtypes indicating the potential of FDG-PET scans in aiding FTD diagnostics. Indeed, use of FDG-PET together with magnetic resonance imaging (MRI) scans can increase the ability to discriminate between Alzheimer’s disease and FTD cases (Dukart et al., 2011). These findings also indicate that glucose hypometabolism may have a role in FTD pathogenesis.

Longitudinal studies concluded there is a continual spread of glucose hypometabolism over time and disease progression (Diehl-Schmid et al., 2007; Jacova et al., 2013; Fukai et al., 2018; Bejanin et al., 2020; Clarke et al., 2021). One longitudinal study followed patients from all clinical subtypes of FTD for 18 months and established differential patterns of glucose hypometabolism spread (Bejanin et al., 2020). Bhv-FTD glucose hypometabolism began in the frontal lobes and spread into neighboring structures, an observation echoed by another longitudinal study on bhv-FTD (Diehl-Schmid et al., 2007; Bejanin et al., 2020). In sv-FTD, baseline glucose hypometabolism was observed in the anterior temporal and medial prefrontal cortex and spread into the temporal, orbitofrontal and lateral parietal cortices (Bejanin et al., 2020). Lastly, in nfv-FTD, glucose hypometabolism originated in the motor cortex and progressively extended into the dorsolateral, dorsomedial, and precentral cortices (*n* = 7) (Bejanin et al., 2020). Familial FTD longitudinal studies provided additional insight by identifying cases before clinical disease onset. These concluded that glucose hypometabolism predates FTD symptoms (Arvanitakis et al., 2007; Jacova et al., 2013; Deters et al., 2014; De Vocht et al., 2020; Clarke et al., 2021). Studies conducted in *PGRN* and P301L *MAPT* mutation carriers found that brain glucose hypometabolism began on average 7 and 12.5 years before disease onset, respectively (Jacova et al., 2013; Clarke et al., 2021). These findings indicate that glucose hypometabolism has a role in FTD pathogenesis, not only accompanying but also predating phenotypic disease onset and progression.

Furthermore, glucose hypometabolism is found to exceed the borders of brain atrophy across FTD types (Nestor, 2003; Desgranges et al., 2007; Morbelli et al., 2016). This, together with the fact that glucose hypometabolism predates disease phenotypes, suggests glucose hypometabolism could at least in part underly neurodegeneration and FTD pathogenesis. This is supported by the finding that pre-symptomatic FTD causing mutant *MAPT* carriers had intermediate levels of brain atrophy compared to symptomatic carriers but comparable glucose hypometabolism (Deters et al., 2014). However, this is not always the case, brain atrophy exceeded the regions glucose hypometabolism in some brain regions in bhv-FTD patients (Buhour et al., 2017), suggesting the relationship could be more complex.

Considering the close coupling of glucose uptake and cerebral blood flow it is unsurprising that in FTD patients, hypoperfusion and glucose hypometabolism co-localize (Mergenthaler et al., 2013; Verfaillie et al., 2015). A longitudinal study with pre-symptomatic *PGRN* and *MAPT* mutation carriers identified hypoperfusion independent of brain atrophy before symptomatic disease (Dopper et al., 2016). Hypoperfusion could be causal for glucose hypometabolism as hypoperfusion would hinder adequate glucose supply. However, the opposite was observed in FTD patients, with glucose hypometabolism extending beyond the borders of hypoperfusion (Anazodo et al., 2018). Since hypoperfusion is not causal for glucose hypometabolism, this raises the question of what causes impaired glucose metabolism.

Studies looking at the metabolic profile of FTD patients revealed dysregulation of glucose metabolism pathways on a more global scale (Ahmed et al., 2014; Murley et al., 2020). When looking at sv-FTD and bhv-FTD patients’, the latter had lower high-density lipoprotein levels as well as increased total cholesterol levels compared to controls and both had increased triglyceride levels (Ahmed et al., 2014). Moreover, both FTD cohorts possessed increased fasting insulin levels, which together with the aforementioned results indicate insulin resistance (Ahmed et al., 2014). Changes in diet for bhv-FTD patients in particular have been documented, with an increased tendency for sweeter foods (Piguet et al., 2011). Nonetheless, changes in diet are unlikely to be the sole cause for insulin resistance, and FTD pathophysiology may underlie the observed changes—a longitudinal study may be able to shed further light on the onset of insulin resistance in FTD patients and its relationship with disease progression (Ahmed et al., 2014). Insulin resistance involvement has been documented in other neurodegenerative diseases and has been proposed to contribute to neurodegeneration by exacerbating inflammatory responses and oxidative stress (Craft and Watson, 2004).

Moreover, A study involving 134 FTD patients analyzed 842 plasma metabolites and identified 49 differently altered metabolites compared to controls among FTD patients, seven were involved in energy production or carbohydrate pathways (Murley et al., 2020). Observations included reduced levels of the glycolysis end product pyruvate, the TCAC intermediate succinate, glycerol-3-phosphate (a molecule pertinent to NADH re-oxidation) and glucogenic amino acids (Murley et al., 2020). Whilst the first three results indicate reduced glucose metabolism activity, the last finding may point to a compensatory mechanism resulting from reduced glucose intake by cells (Wilkins and Trushina, 2018; Murley et al., 2020). Simultaneously, elevated levels of the commonplace polysaccharide’s maltose and maltotetraose were recorded. Furthermore, elevated serotonin levels were found in FTD patients (Murley et al., 2020). Serotonin in the body’s periphery stimulates the secretion of insulin from pancreatic β-cells and inhibits glucose uptake by the liver (El-Merahbi et al., 2015). These results indicate glucose metabolism dysregulation in patients.

Unfortunately, due to the lack of effective *in vivo* tracers for FTD proteinopathies, it is currently impossible to investigate whether glucose hypometabolism overlaps with the accumulation of protein deposits in patients’ brains. This is currently an active area of research, and it is hoped soon there will be tracers with increased specificity and selectivity for FTD pathologies (Tsai et al., 2019).

Collectively both imaging studies and metabolic studies indicate widespread impairment of glucose metabolism across FTD patients, with the brain in particular presenting with glucose hypometabolism. These studies indicated that glucose hypometabolism precedes FTD disease onset, suggesting that glucose metabolism dysregulation may play a role in FTD pathophysiology. Therefore, understanding the mechanism of impaired glucose metabolism could help clarify FTD pathogenesis and aid the search for novel therapeutics.

## Effects of Frontotemporal Dementia Proteinopathies on Glucose Metabolism

Research has begun to focus on identifying mechanistic links between glucose metabolism and FTD pathophysiology. Here we will now review recent research linking glucose metabolism to FTD proteinopathies.

### TAR DNA-Binding Protein 43 Proteinopathy and Glucose Metabolism

TDP-43 proteinopathy is the most common FTD proteinopathy and has been extensively studied, with most studies using ALS linked TDP-43 mutations (as these are more common) to model disease. As these mutations lead to the formation of aggregates similar to those found in FTD patients, these studies are likely also relevant to FTD. However, few studies directly focus on glucose metabolism in TDP-43 proteinopathy (De Conti et al., 2017).

Many studies also focus on understanding TDP-43’s physiological function using loss of function studies, which have highlighted the role of TDP-43 in glucose metabolism. Downregulation of TDP-43 in a variety of human cell lines leads to down-regulation of key glycolytic pathway components. TDP-43 depletion in SH-SY5Y cells leads to significant drops in expression of glycogen phosphorylase (which catalyzes the rate-limiting step in glycogenolysis), and hexokinase-1 (which performs the first step in glycolysis) (Štalekar et al., 2015). In hepatocellular carcinoma, melanoma, and SK-HEP1 cells TDP-43 downregulation leads to a drop in the expression of glycolytic enzymes such as PFKP (phosphofructokinase platelet isoform, the rate-limiting enzyme in glycolysis), ENO2 (encoding enolase 2) and the glucose transporters GLUT1 and GLUT4 (Park et al., 2013; Zeng et al., 2017). This would result in lowered glucose import and glycolysis, as well as a drop in the production of glycogen, a prominent energy store in the brain. These studies, therefore, suggest that TDP-43 plays a fundamental role in sustaining glucose metabolism, and therefore energy availability, by modulating the expression of key components of the pathway.

Conversely, TDP-43 depletion leads to an increase in transketolase, which is involved in the PPP, suggesting a possible re-routing of glucose *via* the PPP, which could affect redox homeostasis (Štalekar et al., 2015). In fruit fly models, over-expression of ALS linked TDP-43 mutation G298S lead to elevated glucose-6-phosphate dehydrogenase expression (an enzyme responsible for committing glucose to the PPP), accompanied by increased PPP activity in motor neurons (MNs) (Manzo et al., 2019). This suggests that glucose metabolism dysregulation occurs downstream of TDP-43 proteinopathy in neurons.

These studies were not carried out in mammalian neurons, therefore the relevance of TDP-43 in the regulation of glucose metabolism in the brain and in a pathological context needs to be confirmed. Studies in model organisms, such as fruit flies, however, go some way to show relevance to a disease context.

In mammalian neuronal models, such as primary rat astrocytes, TDP-43 inclusions lead to a reduction in the expression of the lactate exporter MCT1 (Velebit et al., 2020), which could result in a drop of astrocytic lactate export and therefore lactate availability to neurons. TDP-43 inclusions also led to reduced β2 adrenergic receptor (β2-AR) expression. β2-AR activation leads to increased cyclic adenosine monophosphate (cAMP) and Ca^2+^ cellular concentrations, and consequent upregulation of glucose metabolism (Velebit et al., 2020). Consistently with this, noradrenaline stimulation yielded a reduced and delayed increase in Ca^2+^ and cAMP concentrations, however, lactate production was undisturbed (Velebit et al., 2020).

Additionally, wildtype TDP-43 directly affects mitochondrial metabolism, thus further impacting energy production. Expression in the brain of fruit flies leads to reduced mitochondrial size and increased mitochondrial number (Khalil et al., 2017), whereas over-expression of mutant and wild-type TDP-43 in human embryonic kidney-293 (HEK293) cells and primary cortical rat neurons, leads to abnormal membrane potential followed by mitochondrial fragmentation (Wang et al., 2016). TDP-43 possesses motifs facilitating mitochondrial import and localizes to the inner mitochondrial membrane in FTD patients, mostly to the cristae of cortical neurons’ mitochondria, blocking the targeting of TDP-43 to mitochondria ameliorated the neurodegenerative phenotype of TDP-43 overexpression mouse models (Wang et al., 2016). TDP-43 binds to mRNA transcripts of mitochondrial electron transport complex I subunits, inhibiting their translation (Wang et al., 2016). This may promote complex I disassembly, causing mitochondrial dysfunction (Wang et al., 2016).

TDP-43 might also have more systemic effects on glucose metabolism as TDP-43 loss of function impairs insulin secretion by pancreatic β-cells; this could lead to a global reduction of insulin signaling across the body, including the brain, further exacerbating glucose hypometabolism (Araki et al., 2019). However, it has not been shown if TDP-43 proteinopathy in FTD affects pancreatic β-cells.

**FIGURE 3:**
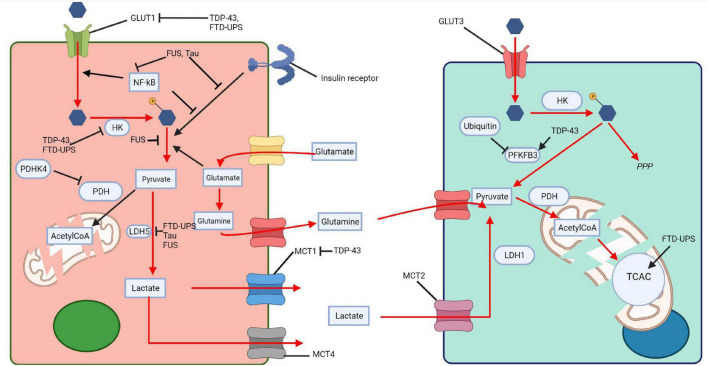
Impaired glucose metabolism across proteinopathies. Tau, TDP-43, FUS, and FTD-UPS affect various steps of glucose metabolism in neurons and glia, thus impairing the ANLS. The pink cell represents a astrocyte and the light green cell a neuron. HK, hexokinase; PDHK4, pyruvate dehydrogenase kinase 4; LDH, lactate dehydrogenase; MCT, monocarboxylate transporter; PDH, pyruvate dehydrogenase; TCAC, tricarboxylic acid cycle; PFKFB3, 6-phosphofructo-2-kinase/fructose-2,6-biphosphatase 3; PPP, pentose phosphate pathway; AcetylCoA, Acetyl coenzyme A. Created with: BioRender.com.

*Drosophila melanogaster* TDP-43 proteinopathy models, over-expressing either wildtype TDP-43 or TDP-43 G298S displayed increased PFK expression, accompanied by an increase in glycolysis, increased glucose consumption and pyruvate production in MNs (Manzo et al., 2019). This was thought to be a compensatory, protective response, possibly in response to a drop in lactate supply from astrocytes, or because of defects in mitochondria metabolism. The protective role of glucose metabolism was indeed confirmed by the fact that over-expression of GLUT3, PFK, and increased sugar feeding extended lifespan and ameliorated disease phenotypes (Manzo et al., 2019). These changes had no impact on TDP-43, indicating that they modulated disease downstream of TDP-43 accumulation (Manzo et al., 2019). Interestingly, *Caenorhabditis elegans* expressing mutant TDP-43 and FUS grown in an enriched glucose environment had reduced protein misfolding, also suggesting that increased glucose metabolism can ameliorate disease associated phenotypes (Tauffenberger et al., 2012). Together this confirms that impaired glucose metabolism has a role in FTD-TDP-43 pathophysiology and that promoting glucose metabolism, albeit in animal models, can ameliorate disease phenotypes. TDP-43 has been extensively shown to impact glucose metabolism and the communication between neurons and astrocytes, potentially explaining the reduction in glucose metabolism seen in PET imaging (Figure 3). The fact that increasing glucose metabolism in some model systems can ameliorate disease goes some way to suggesting that glucose impairments are driving disease progression rather than being consequences of neurodegeneration. This also suggests that glucose metabolism is a viable and interesting therapeutic target.

Some of the genes, and genetic alternations, known to be associated or underly TDP-43 proteinopathy have also been linked to glucose metabolism. *PGRN* is required for the development of insulin resistance in mice fed a high fat diet (Matsubara et al., 2012). Treating cells with a *VCP* inhibitor led to increased TCAC and glycolysis metabolite expression (Parzych et al., 2019). Similar defects in glucose metabolism have been associated with C9 mutations (see below). In summary, genetic mutations associated with TDP-43 proteinopathy have been linked to impaired glucose metabolism on both a cellular and systemic level, with some findings seemingly mirroring those from TDP-43 mutant and overexpression studies.

Most of these studies, however, were conducted in animal models or cell cultures and may not be representative of FTD patients. Research would benefit from conducting co-culture experiments using induced pluripotent stem cells (iPSC) of neurons and astrocytes derived from patients with FTD-TDP-43. Proteomic studies together with studies using labeled glucose could lead to further understanding of how the ANLS is modulated and how this has a pathogenic role.

### C9orf72 Hexanucleotide Repeat Expansion and Glucose Metabolism

C9orf72 hexanucleotide repeat expansion (C9) is the most common genetic cause of sporadic and familial FTD and ALS in populations of European ancestry. The expansion is located in the first intron of the gene C9orf72, the general population has less than 30 copies of the repeat which, in patients, can expand to thousands of copies (DeJesus-Hernandez et al., 2011; Renton et al., 2011; Majounie et al., 2012; Beck et al., 2013). C9 repeats are transcribed into highly stable RNAs which form foci in patients’ neurons (Gendron et al., 2013; Kim and Lee, 2013; Mizielinska et al., 2013), these are translated *via* a non-canonical form of translation called RAN (Repeat Associated Non-atg mediated translation) translation, occurring in all open reading frames, to generate five different dipeptide repeat (DPR) proteins, which also accumulate as aggregates in patients’ brains (Gendron et al., 2013; Lee et al., 2013; Mori et al., 2013; Zu et al., 2013; Cooper-Knock et al., 2014; Mizielinska et al., 2014). The pathogenic mechanism of the expansion is still unclear, with some studies suggesting the stable RNA foci can sequester several important proteins, leading to cellular dysfunction (Sabeti et al., 2007; Kim and Lee, 2013; Cooper-Knock et al., 2014; Balendra and Isaacs, 2018; Swinnen et al., 2018; Frottin et al., 2021), and others suggesting that toxicity is mostly linked to the DPRs translated from the repeats, leading to DNA damage, mitochondrial dysfunction, nuclear-cytoplasmic shuttling defects, a block in general translation and proteasomal dysfunctions amongst others (Mizielinska et al., 2014; Lopez-Gonzalez et al., 2016; Balendra and Isaacs, 2018; Moens et al., 2018, 2019; Choi et al., 2019; Mehta et al., 2021). There is also evidence that the reduced expression of C9orf72 itself, due to the presence of the expansion, might also contribute to disease (Zhu et al., 2020).

Increasingly, there is evidence that C9 expansion might also lead to alterations in glucose metabolism, independently of downstream TDP-43 aggregation, as most of the models used do not present with TDP-43 aggregates. Gene level network analysis of the transcriptome of MNs derived from C9 carrying ALS patients revealed that glucose metabolism associated genes’ expression is almost halved (Cooper-Knock et al., 2015). Moreover, C9 iPSC MNs have reduced expression of mitochondrial electron transport transcripts, underlying dysfunctional oxidative phosphorylation (Mehta et al., 2021). Notably, no changes in glycolysis were recorded (Mehta et al., 2021). However, induced astrocytes derived from C9 patients were found to have a 37.5% loss in metabolic flexibility compared to controls across a panel of 91 potential energy substrates (Allen et al., 2019). Interestingly, loss of glycogen phosphorylase and phosphoglucomutase was recorded in both astrocytes and neurons, resulting in reduced glycogen metabolism (Allen et al., 2019). C9 fly models also have reduced expression of insulin receptor ligands in the brain (Atilano et al., 2021). Intriguingly treating these flies with insulin ameliorated their disease phenotypes, an observation replicated in mammalian cells (Atilano et al., 2021), suggesting that impaired insulin signaling, and potentially glucose metabolism, could be driving toxicity.

### Tau Proteinopathy and Glucose Metabolism

Tau proteinopathy is perhaps the most extensively studied among the FTD proteinopathies, primarily because of the link between hyperphosphorylated Tau and Alzheimer’s disease (AD). Many AD studies for example utilize triple transgenic mice (3xTg) which, among two AD causing mutations in Amyloid Precursor Protein (APP) and Presenilin-1 (PSEN1) also incorporate the P301L Tau mutation, which is causal for familial cases of FTD Tauopathy (Oddo et al., 2003). This means that many AD studies using this model may prove relevant to FTD.

3xTg mouse-derived astrocytes show reduced glycolytic activity and glucose hypometabolism (Dematteis et al., 2020). This is corroborated by the observation that 3xTg astrocytes have lessened intracellular lactate build-up upon MCT inhibition and that L-serine production (derived from the glycolysis intermediate 3-phosphoglycerate) is decreased in 3xTg mice (Douce et al., 2020). Interestingly, 13-month-old 3xTg mice, suffering from extensive Tauopathy, have a 50% reduction in lactate production, whereas 7-month-old mice, with limited Tauopathy, display glucose hypermetabolism in their cortex (Sancheti et al., 2014a,b). This suggests that indeed Tauopathy may drive the reduction in glucose metabolism.

Models of pure FTD, such as human iPSC derived-neurons expressing 10 + 16 *MAPT* mutations have a more significant reduction in ATP production compared to controls when treated with the glycolysis inhibitor iodoacetic acid, also suggesting impaired glucose metabolism (Esteras et al., 2017).

Mitochondrial dysfunction has also been observed in FTD Tauopathies (Delic et al., 2015; Esteras et al., 2017; Britti et al., 2020). Proteomic analysis of P301L *MAPT* mutation carrying mouse models and humans revealed reduced expression of ATP synthase, of subunits of complex I of the electron transport chain and malate dehydrogenase (a protein essential to the malate-aspartate shuttle across mitochondria) leading to higher membrane potentials and reduced complex I function (David et al., 2005; Delic et al., 2015). These findings were replicated in human iPSC derived-neurons expressing 10 + 16 mutant *MAPT*, however, ATP synthase activity was increased, suggesting it might be compensating for complex I’s dysfunctional activity (Esteras et al., 2017). Expression of *MAPT* 10 + 16 mutations in neurons inhibits sodium calcium exchanger leading to a reduced mitochondrial calcium efflux and mitochondrial depolarization (Britti et al., 2020). Since calcium ions facilitate oxidative phosphorylation and ATP production, this would further impede energy production (Griffiths and Rutter, 2009).

Additionally, Tau has been shown to directly regulate insulin signaling in the brain. Tau knock-out mice have impaired IRS-1 tyrosine phosphorylation (required to activate insulin signaling) and increased Ser636 IRS-1 phosphorylation (which inhibits insulin signaling) leading to insulin resistance (Marciniak et al., 2017). Tau also binds and inhibits phosphate and tensin homolog (PTEN), a negative regulator of the insulin signaling pathway. Finally, hyperphosphorylated Tau was found to lose its ability to bind phosphoinositide 3-kinase (PI3K), a downstream kinase in the insulin signaling cascade, however, the physiological implications of this are not known (Reynolds et al., 2008; Marciniak et al., 2017). Together, these findings indicate that Tau loss of function may result in aberrant insulin signaling.

Interestingly, reduced insulin signaling was also found to exacerbate Tau hyperphosphorylation. Mice, expressing human Tau, treated with streptozotocin (a substance which destroys insulin producing cells) experienced PP2A downregulation and Tau hyperphosphorylation—a process reversed by insulin injections (Planel et al., 2007; Gratuze et al., 2017). In accordance, type I diabetic mouse models experienced Tau hyperphosphorylation *via* GSK-3β (a Tau phosphorylating kinase) (Jolivalt et al., 2008). A reduction in glucose metabolism in mice was also associated with increased Tau phosphorylation (Liu et al., 2004; Lauretti et al., 2017), suggesting that glucose hypometabolism could directly exacerbate Tauopathy.

Glutamate homeostasis is also impaired in FTD Tauopathies. Glutamate released by neuronal firing is taken up in astrocytes and stimulates glycolysis, coupling neuronal activity and astrocytic energy supply *via* the proposed ANLS (Pellerin and Magistretti, 1994). Glutamate is converted into glutamine in astrocytes and shuttled to neurons, where it is converted back to glutamante and can be fed into the TCAC further implicating its role in glucose metabolism (Bak et al., 2006). Transgenic mice expressing wildtype or P301L Tau have reduced expression of glutamate transporter 1, responsible for 95% of astrocytic glutamate uptake, coupled with a 28% reduction of total glutamate transport compared to controls (Dabir et al., 2006). This potentially explains why reduced hippocampal glucose metabolism correlated with reduced glutamate homeostasis in P301L Tau expressing mice (Nilsen et al., 2013). Collectively, these findings indicate that impaired glutamate homeostasis may in part underly glucose hypometabolism.

In conclusion, FTD Tauopathies may have a large impact on glucose metabolism. Tauopathy can directly inhibit glycolysis, drive mitochondrial dysfunction, lead to brain insulin resistance and cause disturbances in glutamate homeostasis (Figure 3). However, many of these studies would benefit from being replicated in pure FTD-Tau models, without AD related mutations. Further treatment options exploring upregulation of glucose metabolism should also be investigated.

### Fused in Sarcoma Proteinopathy and Glucose Metabolism

Few studies looking at FUS proteinopathy, directly discuss glucose metabolism. Moreover, most literature analyzing FUS proteinopathy utilizes P525L mutant FUS, a mutation causal for ALS. Although this mutation is not related to FTD, it leads to FUS inclusions, similar to those associated with FTD, making these studies potentially relevant.

HEK293 T-cells and neuroblastoma SH-SY5Y cells treated with exogenous wild-type FUS or mutant P525L FUS have reduced ATP levels compared to controls (Wang et al., 2015). Moreover, cytoplasmic FUS interacts with several essential glycolytic proteins including PFK, pyruvate kinase (PK), phosphoglycerate kinase 1 (PGK), glucose-6-phosphate isomerase (GPI), and α-enolase (Wang et al., 2015). These enzymes could potentially be sequestered by the increased cytoplasmic FUS and FUS inclusions found in FTD, hindering glycolysis. Additionally, wild-type FUS overexpression or mutant FUS expression in transgenic mice or NSC34 cells activates glycogen synthase kinase 3β (GSK-3β), which could potentially lead to phosphorylation of insulin receptor substrate (IRS) 2, and inhibition of insulin stimulated glucose uptake (Stoica et al., 2016). This may further downregulate glucose metabolism, however, it remains to be confirmed whether FUS-mediated GSK-3β activation does indeed result in IRS-2 phosphorylation (Sharfi and Eldar-Finkelman, 2008; Kaidanovich-Beilin and Woodgett, 2011; Stoica et al., 2016).

FUS might also affect glucose metabolism in astrocytes: FUS expression in astrocytes promotes astrocyte reactivity and inflammation, most likely through increased activation of nuclear factor kappa-light-chain-enhancer of activated B cells (NF-κB) (Ajmone-Cat et al., 2019). NF-κB overexpression has been found to decrease insulin sensitivity and thus impair glucose uptake in skeletal muscle, but increases glucose import B-cell lymphomas (Zhang et al., 2010; Sommermann et al., 2011). However, a study elucidating the effect of FTD-FUS proteinopathy on astrocytic glucose metabolism has yet to be done.

Numerous studies have identified mitochondrial damage and impaired mitochondrial ATP production as hallmarks of FUS proteinopathy (Deng et al., 2015, 2018; Stoica et al., 2016; So et al., 2018). FUS can localize to mitochondria, possibly *via* an interaction with HSP60 and, once there, FUS induces the mitochondrial unfolded protein response, promoting HSP60 expression in a potential negative feedback loop (Deng et al., 2015, 2018). Suppressing HSP60 expression ameliorates cellular disease phenotype emphasizing the role of mitochondria in FUS mediated toxicity (Deng et al., 2015). Mammalian neurons, like HT-22 cells, overexpressing wildtype FUS experience mitochondrial cristae loss, increased reactive oxygen species production, disturbed mitochondrial membrane potential, reduced ATP generation and eventual mitochondrial fragmentation followed by cell death (Wu et al., 2011; Deng et al., 2015, 2018). FUS also disrupts the interaction of the endoplasmic reticulum with mitochondria, which is fundamental for mitochondrial Ca^2+^ homeostasis and ATP production (Stoica et al., 2016; Deng et al., 2018). In HEK293 cells, FUS activates GSK3ß thus disrupting mitochondrial protein tyrosine phosphatase interacting protein 51 from tethering endoplasmic reticulum’s vesicle-associated membrane protein-associated protein B (Stoica et al., 2016).

These findings are, however, challenged by the observation of no impaired mitochondrial or glycolytic function in iPSC MNs derived from ALS FUS mutant patients which produce similar FUS proteinopathies as seen in FTD patients (Vandoorne et al., 2019).

At present FUS proteinopathy appears to impact glycolysis. Moreover, FUS proteinopathy may cause mitochondrial dysfunction. However, most studies conducted would benefit from being extended into human or alternate mammalian models to ascertain their validity in FTD.

### Frontotemporal Dementia-UPS Mutation Carriers and Glucose Metabolism

FTD-UPS is, among others, associated with mutations in *CHMP2B* (De Conti et al., 2017). Due to the rarity of this proteinopathy, it is scarcely studied. Nonetheless, two recent studies have begun to unveil the association between glucose metabolism and CHMP2B associated FTD using iPSC from patients with *CHMP2B* mutations (Zhang et al., 2017; Aldana et al., 2020).

Through a transcriptomic and proteomic analysis in iPSC neurons, extensive alterations in glycolysis, the TCAC and the electron transport chain were identified in *CHMP2B* mutation carriers (Aldana et al., 2020). Glycolysis was decreased, with crucial glycolytic enzymes, including hexokinase, being downregulated (Aldana et al., 2020). Moreover, GLUT1 expression was significantly downregulated indicating deficient glucose import (Aldana et al., 2020). In line with this, lactate production was downregulated, by around 15%, compared to isogenic controls (Aldana et al., 2020). Collectively this supports the observed glucose hypometabolism in FTD patients, indicating deficient glucose intake and metabolism in neurons. However, this study solely focused on neurons, it would be interesting to study the effects on astrocytes, especially given the increased glutamate uptake in *CHMP2B* mutant astrocytes, which is known to upregulate astrocytic glycolysis (Pellerin and Magistretti, 1994; Aldana et al., 2020).

Similarly to other FTD linked mutants, *CHMP2B* mutant neurons also display mitochondrial impairments with defective cristae and downregulated oxidative phosphorylation compared to controls (Zhang et al., 2017; Aldana et al., 2020). However, the TCAC was upregulated with the expression of the relevant enzymes increasing and the cycling rate rising by ∼25% (Aldana et al., 2020). This may be a compensatory mechanism. To fuel the TCAC in the absence of glucose, an alternative source is required—potentially provided by glutamate. Supporting this, astrocytes with *CHMP2B* mutations increased their glutamate uptake and phosphate-activated glutaminase, an essential enzyme converting glutamine back to glutamate, was upregulated in neurons (Aldana et al., 2020). However, the enzyme glutamate dehydrogenase, which catalyzes the conversion of glutamate to the TCAC intermediate α-ketoglutarate, was downregulated, suggesting a more complicated picture requiring further research (Aldana et al., 2020).

These studies collectively indicate downregulated glycolysis in *CHMP2B* mutation carrying neurons and impaired mitochondrial function (Figure 3). Astrocytic glucose metabolism in *CHMP2B* carriers remains largely unknown, co-culture experiments would allow monitoring of the role of CHMP2B in both astrocytes and neurons, and in the ANLS.

## Conclusion

In summary, impaired glucose metabolism is extensively observed in FTD patients, and modeling of the underlying proteinopathies, suggests direct modulation of glucose metabolism (Table 3 and Figure 3). Common themes appear to emerge, most proteinopathies lead to reduced glycolysis, defective insulin signaling and impaired mitochondrial function. The concomitant impairment of glycolysis and mitochondrial activity could lead to a substantial energetic deficit in neurons in FTD.

**TABLE 3:** A table presenting a summary of the major findings in the discussed literature.

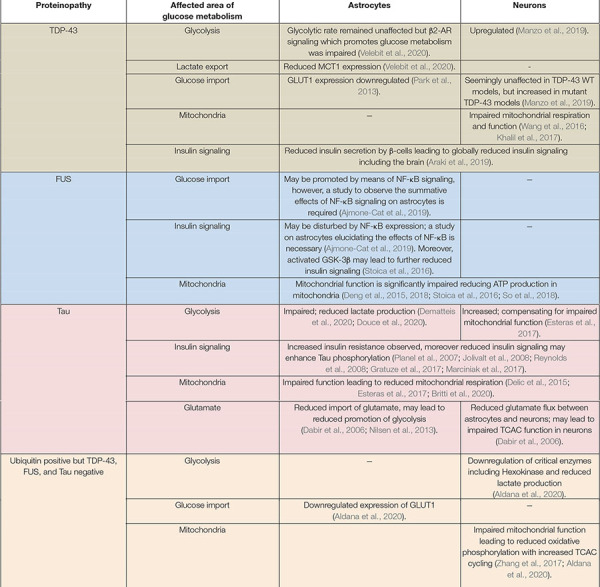

*A summary table of findings regarding disturbed glucose metabolism in the different FTD proteinopathies. “−” indicates an area where specific studies are outstanding. Processes affecting both neurons and astrocytes are presented as merged cells in the table. The different proteinopathies are indicated in the far left column and color coded across the rows.*

However, glucose metabolism dysregulation can also act upstream of the proteinopathies: genetic risk factors (such as C9), can modulate glucose metabolism in the absence of downstream proteinopathy, and a reduction of insulin signalling, can exacerbate Tauopathies, suggesting glucose metabolism dysregulation may act to exacerbate proteinopathy as well as downstream toxicity.

Much, however, remains unknown. The lack of consensus on whether FTD proteinopathies act *via* a gain of toxicity or loss of function model, leads to divergent research models with studies using either overexpression or knockout models, sometimes resulting in opposing conclusions. Research also uses diverse organisms to model FTD proteinopathies ranging from insects to mammalian animal models or cell cultures, at different developmental stages, which are known to affect metabolism. Identifying the most relevant disease model can be challenging.

To further understand how FTD affects glucose metabolism, and in particular the interplay between astrocytes and neurons, research would greatly benefit from co-culture experiments with patient-derived cells.

However, the finding that promoting glucose metabolism in FUS and TDP-43 proteinopathies ameliorates disease phenotypes indicates that glucose metabolism could be a driver of FTD pathophysiology and therefore could become a promising therapeutic target.

## Author Contributions

LG and TN wrote the manuscript. Both authors contributed to the article and approved the submitted version.

## Conflict of Interest

The authors declare that the research was conducted in the absence of any commercial or financial relationships that could be construed as a potential conflict of interest.

## Publisher’s Note

All claims expressed in this article are solely those of the authors and do not necessarily represent those of their affiliated organizations, or those of the publisher, the editors and the reviewers. Any product that may be evaluated in this article, or claim that may be made by its manufacturer, is not guaranteed or endorsed by the publisher.
